# Oocyte cryopreservation in the setting of a vascular endothelial growth factor (VEGF)-producing paraneoplastic syndrome: a case report and review of literature

**DOI:** 10.1186/s40738-020-00086-z

**Published:** 2020-10-28

**Authors:** Emily Spurlin, Paula Brady

**Affiliations:** 1 Department of Obstetrics and Gynecology, Columbia University Irving Medical Center, 622 West 168th St., New York, NY 10032 USA; 2 Department of Obstetrics and Gynecology, Columbia University Fertility Center, Columbia University Irving Medical Center, 5 Columbus Circle, Penthouse, New York, NY 10019 USA

**Keywords:** Controled ovarian hyperstimulation, Oocyte cryopreservation, Oncofertility, POEMS paraneoplastic syndrome, Ovarian hyperstimulation syndrome

## Abstract

**Background:**

Many reproductive aged women with new oncologic diagnoses choose to undergo emergency oocyte or embryo cryopreservation prior to initiating potentially gonadal toxic oncologic therapies. Ovarian hyperstimulation syndrome (OHSS) is a potential complication of these treatments and can be particularly dangerous in these patients due to their underlying medical illness and by delaying lifesaving oncology treatment. This case report details a multipronged approach to OHSS prevention in a patient with a paraneoplastic syndrome defined by overproduction of vascular endothelial growth factor (VEGF), which is also primarily responsible for OHSS.

**Case presentation:**

A 29 year old nulligravid woman was diagnosed with a rare multisystem paraneoplastic syndrome (Polyradiculoneuropathy, organomegaly, endocrinopathy, monoclonal plasma cell disorder and skin changes, known as POEMS) and presented for fertility preservation consultation prior to autologous stem cell transplant. She successfully underwent oocyte cryopreservation without complications due to aggressive OHSS prophylaxis with both a dopamine agonist and aromatase inhibitor during and after stimulation and a gonadotropin releasing hormone agonist (GnRH-A) for final oocyte maturation, without delay in her subsequent oncology treatment.

**Conclusions:**

This is the first report of a patient with POEMS undergoing controlled ovarian hyperstimulation (COH). Oocyte cryopreservation was successful and without complications due to a combination of prophylactic measures against OHSS (cabergoline, letrozole and GnRH-A trigger) and close collaboration between reproductive endocrinology and oncology. This case demonstrates the use of combined measures in targeting VEGF overproduction and OHSS risk during COH.

## Background

Polyradiculoneuropathy, organomegaly, endocrinopathy, monoclonal plasma cell disorder, and skin changes (POEMS) is a rare multisystem paraneoplastic syndrome stemming from monoclonal proliferation of plasma cells. Prominent features also include sclerotic bone lesions, hematologic abnormalities, and elevated vascular endothelial growth factor (VEGF) levels leading to increased vascular permeability, signs of which include papilledema, ascites and extravascular volume overload [1]. Diagnostic criteria for the syndrome are shown in Table 1. POEMS is also associated with hypogonadism in men, but reproductive and endocrine effects have not been sufficiently studied in female patients due to the rarity of POEMS and age at diagnosis commonly beyond the reproductive years [2]. Prevalence of POEMS is estimated at 0.3 per 100,000, and approximately 40% of patients are female [3]. The median age of onset of is 54 years, and 84% of affected patients are over age 65 years [3–7].

**Table 1:** Mandatory, major, and minor criteria of POEMS syndrome^a^

**Mandatory Criteria** *(required for diagnosis)*	
♦ Peripheral polyneuropathy	
♦ Monocolonal plasma cell disorder	
**Major Criteria** *(one required for diagnosis)*	
♦ Osteosclerotic bone lesions	
♦ Elevated VEGF levels	
♦ Castleman disease (angiofollicular lymph node hyperplasia)	
**Minor Criteria** *(one required for diagnosis)*	
♦ Endocrine abnormalities	
○ Adrenal	
○ Thyroid	
○ Pituitary	
○ Gonadal	
○ Parathryoid	
♦ Skin changes	
○ Hyperpigmentation	
○ Hypertrichosis	
○ Glomeruloid hemangiomata	
○ Plethora	
○ Acrocyanosis	
○ Flushing	
○ While nails	
♦ Organomegaly	
○ Splenomegaly	
○ Hepatomegaly	
○ Lymphadenopathy	
♦ Extravascular volume overload	
○ Edema	
○ Pleural Effusion	
○ Ascites	
♦ Thrombocytosis/polycythemia	
♦ Papilledema	

^a^Dispenzieri A. POEMS syndrome: 2017 Update on diagnosis, risk stratification, and management. Am J Hematol. 2017;92:814–29

Treatment consists of radiation and/or systemic therapy based on the extent of bone marrow involvement. Isolated bone lesions are treated with radiation therapy while disseminated bone marrow involvement requires systemic therapy with chemotherapy and stem cell transplantation [1]. Ten-year survival is estimated at 93% [3].

Oocyte or embryo cryopreservation is a standard of care for fertility preservation in patients facing gonadotoxic therapies, including chemotherapy and radiation [8]. This is the first report of fertility preservation in a patient with POEMS, in which a 29 year-old woman underwent controlled ovarian hyperstimulation (COH) and oocyte cryopreservation immediately prior to autologous stem cell transplant with special consideration given to the prevention of ovarian hyperstimulation syndrome (OHSS), due to its shared pathophysiology with POEMS via VEGF.

## Case presentation

The patient is a 29-year-old nulligravida who initially presented with a constellation of symptoms that developed over 1 year, most prominently polyneuropathy with numbness in her hands and feet. She also reported a 20-pound weight gain with abdominal distention and bloating despite a vigorous exercise regimen, joint pain, rash on bilateral upper extremities, and oligomenorrhea. During this time, an endocrinologist diagnosed her with hypothyroidism based on a thyroid stimulating hormone level of 5.3 mIU/mL with negative anti-thyroglobulin and thyroperoxidase antibodies. As part of her assessment of peripheral neuropathy by a neurologist, a serum protein electrophoresis was ordered, which showed a monoclonal gammopathy of unclear etiology. Routine ophthalmology evaluation revealed left eye papilledema on fundoscopic exam initiating an immediate referral to the emergency department for further evaluation.

Following an extensive inpatient assessment, including head magnetic resonance imaging and lumbar puncture, the patient was given the presumptive diagnosis of idiopathic intracranial hypertension and was discharged on acetazolamide therapy. Further outpatient assessment revealed polycythemia with a hemoglobin level of 16.7 g/dL and a VEGF level of 409 pg/mL, four times the upper limit of normal. Computed tomography of the chest, abdomen and pelvis showed multiple diffuse lytic bone lesions as well as hepatomegaly. Sacral bone biopsy revealed neoplastic plasma cells. Due to these many clinical findings, the patient was given a diagnosis of POEMS (Table 1). As the bone lesions were diffuse, the patient was not a candidate for localized radiation and was counseled for autologous stem cell transplant, as well as for fertility consultation.

During her fertility evaluation, the patient’s antimullerian hormone level was 3.6 ng/mL, with antral follicle count of 18. Oocyte and tissue cryopreservation were discussed; the latter is not currently recommended for hematologic malignancies given concerns for reintroducing disease in the future through auto-transplantation [9]. The patient elected to undergo oocyte cryopreservation, and began her random start stimulation in the late follicular phase, with an 18 mm lead follicle, estradiol of 129 pg/mL, and progesterone 0.4 ng/mL. She began stimulation with 150 international units (IU) of recombinant follicle stimulating hormone (FSH) and 150 IU of purified urinary human menotropins, but by cycle day three, this was reduced to 75 units each due to acute moderate edema of the hands and feet. A gonadotropin-releasing hormone antagonist (GnRH-A) was started at the outset of stimulation due to the advanced follicle size. On cycle day four, laboratory assessment and physical examination by the patient’s oncologist were stable, the estradiol level was 84 pg/mL, and the FSH dose was increased to 112 IU per day.

For prevention of OHSS, the patient was prescribed an aromatase inhibitor (letrozole 5 mg per day) and a dopamine agonist (cabergoline 0.5 mg per day) at the outset of her stimulation. On the advice of her oncologist, she also administered daily prophylactic low molecular weight heparin (enoxaparin 40 mg per day). On cycle day eight, with a peak serum estradiol of 177 pg/mL (suppressed by letrozole), three follicles measured 18 mm or greater (19, 22 and 24 mm), and the decision was made to proceed to oocyte retrieval based on follicle diameter. The patient administered a single 2 mg (40 unit) dose of GnRH agonist leuprolide acetate, after which her enoxaparin was held until 24 h after retrieval. During an uncomplicated oocyte retrieval 36 h after trigger injection, seven eggs were retrieved, with four mature and cryopreserved. The enoxaparin, letrozole, and cabergoline were continued for 1 week after retrieval, until the patient’s menses began. Over the course of her cycle, the patient complained of moderate extremity edema and her laboratory values and clinical symptoms of POEMS remained stable. The patient was admitted for autologous stem cell transplant 2 weeks following retrieval, as planned.

## Discussion and conclusion

This is the first report of COH in a patient with POEMS syndrome. The clinical syndromes of POEMS and OHSS are both charterized by VEGF overproduction, and this patient was presumed to be at a significantly higher risk of OHSS during and following COH given her underlying disease.

This patient possessed all the baseline characteristics associated with elevated OHSS risk: age under 35 years, low body mass index (22 kg/m2), and elevated AMH (> 3.36 ng/mL) [10]. In OHSS, increased concentrations of VEGF are released from luteinized granulosa and endothelial cells in response to hCG [11]. VEGF, though interactions with its receptor on endothelial cells, causes increased capillary permeability by altering endothelial junction proteins leading to an extravasation of protein-rich fluid from the vasculature to the extravascular space. This results in the findings typically seen in OHSS, ascites, pleural effusions, electrolyte imbalances, acute kidney injury, and hemoconcentration that can lead to vascular thromboses. Given this patient’s particularly high risk for these complications, multiple methods of OHSS prevention were employed in tandem. While symptoms of OHSS generally peak post-retrieval, VEGF levels have been shown to rise during COH, and for this reason, prophylaxis began during her stimulation [12].

Cabergoline is a dopamine agonist that reduces VEGF-related vascular permeability, in part by reducing VEGF receptor-2 phosphorylation [13]. Dopamine agonists initiated at the time of trigger with GnRH-A have been shown in multiple randomized trials to reduce the risk and severity of OHSS [10]. In order to curtail the treatment-related VEGF rise as much as possible, cabergoline was initiated at the same time as gonadotropin injections in this patient.

Letrozole, an aromatase inhibitor, was also used throughout the patient’s treatment for OHSS prevention. This medication is most commonly used during COH in patients with estrogen-receptor positive malignancies [14]; however, recent evidence suggests letrozole may also reduce the risk of OHSS. A mouse model showed equal efficacy of cabergoline and letrozole in curtailing OHSS physiology [15], and a small randomized trial of 51 women with polycystic ovarian syndrome found a five-fold reduction in OHSS in women receiving letrozole during COH with an antagonist protocol and GnRH-A trigger (though details of letrozole duration were not provided) [16]. Letrozole was also shown to be more effective than aspirin in OHSS prevention in high responders (> 25 eggs or serum estradiol 5000 pg/mL) when started at retrieval for 5 days [17]. The effect of letrozole on OHSS pathophysiology is unknown, as VEGF levels may be higher in patients receiving letrozole during COH; instead, the luteolytic effects of letrozole may accelerate resolution of OHSS symptoms [15, 17].

The use of a GnRH-A for final oocyte maturation is a widely-accepted measure for OHSS prevention. When human chorionic gonadotropin (hCG) is used instead as the trigger method, hCG binds to luteinizing hormone (LH) receptors in the follicle, prompting final egg maturation and supporting corpus luteum function, which includes producing large quantities of VEGF. The GnRH-A results in an endogenous LH surge with the same effect, but with a much shorter half-life (< 60 min, as compared to approximately 24 h for hCG). This short half-life of endogenous LH leads to more rapid luteolysis and lower VEGF levels released, without compromising final oocyte maturity [10, 18–20].

Due to the risk of thrombosis associated with POEMS syndrome, in consultation with the patient’s oncology team, the decision was made to initiate daily prophylactic low molecular weight heparin with stimulation [1, 21]. Little data is available regarding risk of thromboembolism during oocyte or embryo cryopreservation alone (i.e. without fresh embryo transfer), but concern for this complication is supported by the observation that risk of venous thromboembolism is significantly higher in the first trimester of pregnancy following in vitro fertilization and fresh embryo transfer (and 100-fold when treatment was complicated by OHSS), as compared to spontaneous pregnancies [22]. Aspirin was also considered for this patient, which may reduce the risk of OHSS as well as thromboembolism [9], but the decision was made to proceed without aspirin due to concern for hemostasis during oocyte retrieval.

This is the first report of COH and oocyte retrieval attempted in a patient with POEMS. Oocyte yield was low considering the patient’s age and ovarian reserve; this was likely primarily the result of low gonadotropin dosing, limited by early symptoms of OHSS. Even with a modest yield, quality is expected to be optimal given her age. Despite very high risk of OHSS and cancellation, through close collaboration between reproductive endocrinology and oncology, this patient was safely ushered through her fertility preservation treatment. Regular evaluations by her oncology team for close monitoring for POEMS signs and symptoms were important safety measures during the process in addition to monitoring for medication side-effects. Letrozole and carbergoline are typically well tolerated, though it is worth noting that commonly reported side effects of hot flashes, fatigue and dizziness for letrozole, and headache, postural hypotension, nausea, and fatigue for cabergoline were not observed in this case [13, 23, 24]. The combination of multiple OHSS prevention techniques described here – cabergoline and letrozole throughout her stimulation with a GnRH-A trigger – is novel, and allowed for successful oocyte cryopreservation without delay to her bone marrow transplantation.

## Data Availability

In order to protect patient confidentiality, the data generated and/or reviewed during the above publication are not publically available but available from the corresponding author by reasonable request.

## Abbreviations

ART: Assisted reproduction technology
COH: Controlled ovarian hyperstimulation
FSH: Follicle stimulating hormone
GnRH-A: Gonadotropin releasing hormone agonist
HCG: Human chorionic gonadotropin
LH: Luteal hormone
OHSS: Ovarian hyperstimulation syndrome
POEMS: Polyradiculoneuropathy, organomegaly, endocrinopathy, monoclonal plasma cell disorder, and skin changes
VEGF: Vascular endothelial growth factor
